# A systematic review and meta-analysis on the effects of physically active classrooms on educational and enjoyment outcomes in school age children

**DOI:** 10.1371/journal.pone.0218633

**Published:** 2019-06-25

**Authors:** Chloe Bedard, Laura St John, Emily Bremer, Jeffrey D. Graham, John Cairney

**Affiliations:** 1 Department of Health Research Methods, Evidence, and Impact, McMaster University, Hamilton, Ontario, Canada; 2 INfant and Child Health (INCH) Lab, Department of Family Medicine, Master University, Hamilton, Ontario, Canada; 3 INfant and Child Health (INCH) Lab, Faculty of Kinesiology and Physical Education, University of Toronto, Toronto, Ontario, Canada; 4 Faculty of Kinesiology and Physical Education, University of Toronto, Toronto, Ontario, Canada; University of Kentucky, UNITED STATES

## Abstract

**Objectives:**

Despite the relationship between physical activity (PA) and learning outcomes, the school system has not been able to support the inclusion of PA throughout the day. A solution to this problem integrates PA into the academic classroom. The objective of this review is to determine the impact of active classrooms compared to traditional sedentary classrooms on educational outcomes of school-aged children.

**Design:**

We searched ERIC, PubMed, PsychINFO, and Web of Science, reference lists of included studies for randomised controlled studies. Independent reviewers screened the texts of potentially eligible studies and assessed the risk of bias. Data were pooled using random-effects models on standardized mean differences.

**Results:**

This review identified 25 studies examining educational outcomes, including approximately 6,181 students. Risk of bias was assessed as either some or high risk of bias for most of the studies and outcomes. Pooled data from 20 studies and 842 participants measuring academic performance shows a small positive effect of active classrooms compared with traditional, sedentary classrooms (SMD = 0.28, 95% CI: 0.09 to 0.47).

**Conclusions:**

Physically active classrooms may slightly improve academic achievement compared to the traditional sedentary lessons. Future research is needed to ensure that studies are adequately powered, employ appropriate methods of randomization, and measure a wide range of important student outcomes across the full spectrum of the school-age.

## Introduction

The health benefits of physical activity (PA) to children and youth are widely documented [1]. Yet, the prevalence of inactivity in the youth population is high,[2] suggesting most children and youth are not able to realize these benefits. Efforts to increase PA have focused on the school setting, given the fact that the majority of children and youth are in this setting for large periods of the day and week. While this makes practical sense, the objective of increasing PA is often at odds with the scholastic objectives of education administrators and teachers. As such, there has been a push from academic researchers and others to emphasize not only the health benefits of PA and exercise, but also its effects on learning. Participation in PA has been linked to improved cognitive function in a wide variety of measures across childhood and adolescence [3,4]. However, despite the relationship between PA and physical health and learning outcomes, the school system has not been able to adapt their daily structure to support the inclusion of PA to a level (e.g., 60 minutes per day) that supports both positive health and learning outcomes. Children spend two thirds of their day sitting and most of this time is spent in class, with only a few brief periods of respite during recess [5]. With children and youth spending a substantial amount of time in school each day (approximately 8.5 hours each day) over at least 6 months of the year,[6] the opportunities for PA are strictly limited. It is critical that opportunities for physical activity throughout the school day are maximized through initiatives of extending and enhancing existing PA time and/or replacing traditional sedentary time with PA [7]. School-based PA intervention studies have largely focused on extending or enhancing existing PA opportunities (i.e. physical education class) and have been marginally successful,[8] however, many barriers to implementation remain problematic. Teachers still feel pressure from parents, schools, and school-administrators, to prioritize time spent learning core academic content rather than devoting time to PA [9–11]. Naylor and colleagues [12] reviewed 29 studies of school-based physical activity interventions and found that factors relating to time, such as preparation time, teacher overload, and competing demands for other curricular priorities were consistently reported as barriers to successful implementation. This is particularly concerning given the positive relationship between implementation effectiveness and positive health outcomes [12,13].

A feasible, innovative solution to this problem has emerged in recent years and it integrates physical activity into the academic classroom [14]. This teaching technique intentionally blends physical activities into core-content academic lessons to satisfy both the learning outcomes of the classroom without sacrificing time spent engaging in PA, often referred to as movement integration or active classrooms. For example, a mathematics class might have students perform jumping jacks or hop to indicate their answer to a question posed by a teacher. This is distinct from active breaks, which introduces PA without integrating academic content, or active transitions, which involves the use of PA to move from one topic or task to another [15]. This type of intervention has the potential to have superior effects on educational outcomes compared to traditional sedentary lessons through both neurobiological and neurocognitive mechanisms. There is evidence that PA can impact cognitive performance in many pathways depending upon the nature of the activity [16]. In a review on the effect of PA on cognitive functions in children, Best [16], synthesized the experimental literature and found that acute aerobic exercise can induce generalized cognitive improvements through increased blood flow and neurochemical responses leading to upregulation of neurotrophins. The review also found that chronic exercise has been shown to produce morphological changes in brain centres association with learning [16]. Best also found potentially larger effects resulting from studies evaluating cognitively-demanding exercises and posited the following hypothesized mechanism: When an exercise has increased cognitive demands, prefrontal-dependent neural networks, those used during cognitive functions tasks, are activated, and this may lead to enhanced prefrontal neural functioning in tasks that follow this type of exercise. Therefore, since physically active academic lessons could be considered a form of cognitively-demanding exercise, it is possible that these lessons may involve increased activation within these prefrontal networks which may lead to better scores on academic or cognitive tests. Diamond and Ling [17] emphasize in their review of interventions to improve cognitive functioning in children, that programs will be more successful if they also support psychosocial mechanism that have been linked to enhanced cognitive functioning, such as feelings of joy. Students may find physically active lessons more enjoyable compared to their sedentary lessons, and therefore their mood or affect may be positively affected. These positive feelings may enhance their performance on educational or cognitive tests or assessments.

A systematic review was completed by Norris et al. in 2015 [18] on this topic, however due to the small number of studies (n = 6) evaluating the effect of active classrooms on academic achievement, a meta-analysis could not be performed. Qualitatively synthesized results from Norris et al. suggest there is a positive association between active classrooms and learning outcomes [18]. Narrative syntheses, however, have only limited value in the decision-making process. Since this review was published (and, more importantly, since the search was completed) many new randomized trials have been conducted. There is a need to update the review and include a quantitative estimate of the effect to provide decision makers (i.e., educators) guidance on effective teaching practices that can benefit their student’s academic performance. There have been other similar systematic reviews conducted [19], however they have combined studies of different designs and types of classroom-based physical activities such as active breaks and physically active lessons. These reviews are relevant, however they cannot describe the specific effects of classrooms integrating physical activities with academic content. Therefore, the purpose of this study was to conduct systematic review and meta-analysis that asks the following research question: among school-aged children ages 3 to 18 years (preschool to high school), what is the effect of physically active lessons compared to traditional lessons on academic performance?

## Methods

### Criteria for considering studies for this review

#### Population

Children attending any type of school including preschool, primary, middle, or high school; this encompasses children between the ages of 3 to 18 years. We excluded studies that enrolled post-secondary classrooms. Eligibility of the studies was determined by their setting rather than an age criterion: for example, studies that evaluate the intervention in secondary schools but include youth 18 years or older would be included; however, studies that evaluate an intervention in post-secondary school settings and enrol participants under the age of 18 years would be excluded. This criterion is based on setting to ensure that the review did not exclude studies set in high school that included students ages 18 years. Furthermore, this ensured that the interventions would all take place in an elementary school setting and thus be similar enough to combine into a single analysis; a post-secondary school day is operationally different from an elementary school day therefore the effect of an intervention may be different. Studies in which the target population are students with learning disabilities or in special education classrooms were excluded, as the both the physical activity and magnitude of effect would likely be different in these children compared to their typically developing peers. However, no studies were eliminated exclusively based on this criterion.

#### Intervention

The intervention is physically active school lessons in which academic content (e.g., mathematics, geography, language, history, etc.) are taught through physical activities. This could include lessons where the integrated physical activity is either related to the academic content (e.g. if learning the meaning of the word ‘fly’, children would run and move their arms to act out the word [20]) or unrelated to the academic content (e.g. jumping in place to indicate the answer to a math problem). These active lessons are distinct from classroom activity breaks, in which a traditional academic lesson is broken into segments separated by shorts bouts of PA. We were not interested in classroom activity breaks because we wanted to evaluate interventions that did not take away teaching time. We did not place restrictions on the type of academic content being blended with PA, the lesson duration or frequency, or length of the intervention implementation period. However, in cases where the intervention length exceeds one school year and measurements are collected at each year, the evaluation at the end of year one was extracted. This decision serves to minimize the heterogeneity of the comparisons to interventions of shorter duration and eliminate the confounding effects of a summer break on outcomes; however, this decision was made post-hoc after screening (prior to data extraction) when we observed great variability in intervention lengths. We excluded studies in which active lessons were one component of a multi-component school-wide intervention, for example implementing movement integration in the classroom in addition to active transitions, extended physical education classes, and active homework, because change in outcomes cannot be attributed to a single component of the intervention.

#### Comparator

We included studies that compared active lessons to traditional, sedentary academic lessons without the integration of PA. Studies comparing active lessons to activity breaks were excluded.

#### Outcomes

The two primary outcomes are academic performance and cognitive function and secondary outcomes include measures of attention in class and ratings of enjoyment. Academic performance may be measured through provincial/state or standardized tests or class-specific grades. A priority for standardized measures of academic performance was taken over non-standardized test scores when studies presented both Direct measures of cognitive ability or EF (i.e. cognitive flexibility, working memory, inhibition, and fluid intelligence) may be measured through age-appropriate validated tests for children (e.g. Erickson flanker task, list-sorting task, etc.) [21]. Attention in class, operationalized as “time on task”, was assessed with structured direct observation techniques (i.e. momentary time sampling) to compute the proportion of time spent engaged in the assigned task out of the total time observed. Student satisfaction or enjoyment was typically measured using researcher-designed questionnaires or single-items. To avoid issues of multiple comparisons/testing we only analysed each outcome measure at one time point, immediately after the intervention was completed.

#### Design

To ensure this evidence synthesis is based upon the highest quality of evidence, we included studies which randomly assigned individuals or clusters to an intervention or control condition. We also only included studies that were published in English.

### Search methods for identification of studies

As this is an update of the educational outcomes assessed in the Norris et al. [18] review, we evaluated all 11 studies from the Norris et al. [16] review for inclusion in the current review and subsequently included the only two randomized controlled trials that evaluated the relevant outcomes. Then we used the same search strategy and applied date limits covering the time since the Norris et al. [16] search was conducted (March 2014) up to March 3, 2017 to retrieve new studies published since the original review search was conducted. We searched four education-relevant databases: ERIC (ProQuest; date: After March 01 2014); PubMed (date: March 01 2014 to March 03 2017); PsychINFO (limit: 2014-current); and Web of Science (Timespan = 2014–2017). Search terms included physical activity or activit* or exercise (title and abstract), class* or lesson* or learning* (title and abstract), and child* or young*; there were no publication format restrictions. To update the review, the search was repeated on December 3, 2017, and again on February 5, 2019. Specific search terms were modified to conform to the unique requirements of each database. Please refer to Figures A-F in S1 File for examples of our search strategy. Reference lists of included papers were searched as well as grey literature in the following organizational websites:

Play England: http://www.playengland.org.uk/
Active Living Research: http://activelivingresearch.org/
Active Academics: http://www.activeacademics.org/
Institute of Education, University of London: http://www.ioe.ac.uk/index.html.

### Data collection and analysis

#### Selection of studies

A team of four independent reviewers screened the titles and abstracts of retrieved records for possible inclusion so that each record had two independent reviews. Of those identified as possibly eligible, the full-texts were obtained and two independent reviewers assessed the records for inclusion. The 11 studies identified from the Norris et al. [16] review were also screened for eligibility. Disagreements were resolved through discussion and consensus. Study authors were contacted for more information if the full-text was unavailable or details were insufficient to determine eligibility.

#### Data extraction and management

For each included study, one reviewer extracted data into a Microsoft Excel data collection form. Data was extracted on: the characteristics of the study design (including measurement timing); cluster and individual characteristics (by intervention group); intervention characteristics including session duration, frequency, length, academic content type, teacher training, and implementation methods; and types and methods of outcomes measured including the specific instrument tool, a range of possible scores (if reported), and the results for each group at each time point. A second reviewer checked the data for errors and discrepancies were resolved through discussion and consensus.

#### Assessment of risk of bias in included studies

Risk of bias in the included studies was assessed by two independent reviewers using the Cochrane Risk of Bias Tool (RoB 2.0) with the additional considerations for cluster-randomized trials [22]. Specifically, the RoB 2.0 tool is designed for assessing the ‘effect of assignment to an intervention.’ Risk of bias that arises from the randomization and allocation process, deviations from the intended interventions, missing outcome-level data, measurement of the outcomes, and outcome reporting were assessed for each outcome of interest in all included primary studies. Signalling questions within each domain with response options of ‘yes/probably yes’, ‘no/probably no’, and ‘no information’ were used to generate domain-specific judgements of either low risk, some concerns, or high risk of bias. Domain-specific risk of bias assessment were used to judge the overall risk of bias for each study. Disagreements between reviewers were resolved through discussion and consensus.

#### Measures of treatment effect

Outcome measures of the included studies were all continuous and reported on different scales, therefore the standardized mean difference (SMD) and standard error (SE) were used to summarize estimates of effects from individual studies [23]. The magnitude of the standardized mean differences were interpreted using Cohen’s conventions for small (SMD = 0.2), medium (SMD = 0.5), and large (SMD = 0.8) [24]. Generally, studies reported at least two measurements of the outcome (pre and post-intervention) however data on the variability (standard deviation, SD) of the change score was rarely reported; therefore as recommended in the Cochrane handbook,[25] a comparison of the final measurements was used in the absence of a change score and associated SD, thereby eliminating the need to rely on imputed values of the SD of the change score. This imposes an assumption of balanced baseline measurements; however, this issue is controlled for when taking into account risk of bias due to randomization. The final measurement (i.e., post-intervention) and SD for subject-specific academic measures, fluid intelligence, attention, and enjoyment were all entered and directly compared between the active classroom and traditional classroom groups as SMDs. For outcomes of overall academic performance and EFs, trials typically measured and reported multiple scores of equal importance on all participants (e.g., math and spelling test scores; inhibition and working memory); in order to pool all available data without violating the rule of independence, a combined effect (a mean SMD and standard error (SE)) within each outcome was calculated for each study and the generic inverse variance method was used to generate a pooled summary across studies [26]. For example, if the study assessed both math and spelling scores, the SMD would be computed for both and the mean of the two would be entered into the meta-analysis. Lower scores on executive function tests often reflect better performance (e.g. interference effects, response time), therefore when computing an average executive function score these values were multiplied by negative 1 in order to properly account for their reverse value. Outcomes reported on the same scale were meta-analysed as a mean difference between groups on their post-intervention scores.

#### Unit of analysis issues

Data from trials that randomized clusters of schools or classes were adjusted for clustering using the design effect method outlined in Higgins 2011, Chapter 16.3 [25]. In Microsoft Excel, using an appropriate intra-cluster correlation coefficient (ICCs) and the average cluster size, a design effect was calculated for each study and applied to the sample size in each group to compute an effective study sample (always rounded up to the nearest whole number). School-level ICCs were not calculated or reported by any trial author, however the Donnelly 2013 protocol paper [27] estimated an ICC = 0.1 for academic achievement; this value was imputed for all trials that randomized by school. Fedewa et al. [28] was the only study that reported a class-level ICC (0.22) and this was imputed for all studies that randomized by class and childcare centre. When studies reported appropriate mixed effects modelling procedures, they often included time points beyond the scope of the research question and the coefficients were reported as an unstandardized beta therefore we could not statistically combine the data with our other estimates of effect. Therefore, we used the post-intervention score and adjusted the sample size using the procedures described above.

#### Assessment of heterogeneity

Inconsistency between study estimates was both visually and statistically examined, through inspection of the forest plots and consideration of the X^2^ test and I^2^ value, respectively. Thresholds as recommended by the Cochrane handbook,[25] in chapter 9.5.2, were used to interpret I^2^ values.

#### Data synthesis

The random-effects model was used to generate meta-analytic estimates of effects for each outcome using RevMan software. Forest plots of the main analyses and tables containing the results of the sensitivity analyses were also generated. A funnel plot was generated for meta-analyses containing more than 10 studies.

#### Subgroup and Sensitivity analyses, investigation of heterogeneity

Some evidence suggests that preschool children may show larger cognitive improvements than primary and high school children in response to exercise [29]. Therefore, grades were used to define subgroups: preschool (ages 3 to 4 years); primary school (kindergarten to grade 5; ages 5 to 10 years); middle school (grades 6 to 8; ages 11 to 13 years); high school (grades 9 to 12; ages 14 to 18 years). For any sample to be considered within a specific grade category, at least 80% of the sample must be within the age group; in the absence of this information, the mean age of the sample was used to determine the appropriate subgroup category. Sensitivity analyses investigated the impact of varying ICC values for both school- and class-level clusters as well as risk of bias due to randomization and imbalanced baseline scores.

## Results

### Results of search

**Fig 1:**
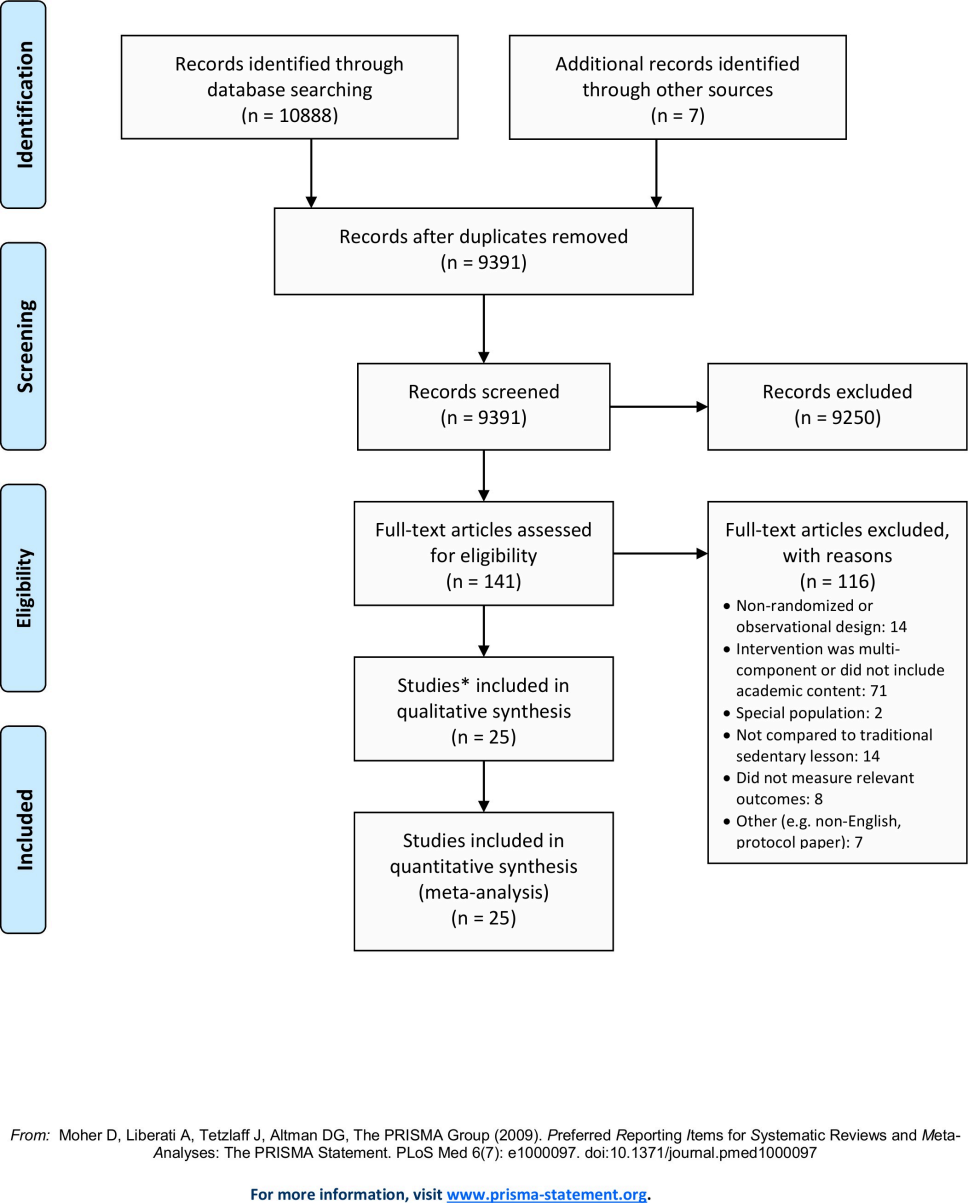
PRISMA flow diagram. *Two studies were published in 4 articles.

The search yielded 10,888 records from all databases; an additional 7 records were identified from organizational websites. We screened 9391 after removing duplicate records. This first level of screening identified 141 full-text articles to be reviewed for eligibility. Of the remaining 141 articles, 23 studies (25 records; two studies each were published in two separate articles) were included in the review. Therefore, in addition to the two randomized controlled trials evaluating educational outcomes identified in the Norris et al.[18] review, a total of 25 studies are included in this review (See Fig 1. for PRISMA flow diagram).

### Included studies

The 25 studies included approximately 6181 students (not adjusted for clustering) in preschool (ages 3 to 4 years; 6 studies), primary school (kindergarten to grade 5; ages 5 to 10 years; 18 studies), and middle school (grades 6 to 8; ages 11 to 13 years; 1 study) schools across Europe, United States of America (USA), and Australia. There were no studies of high school students. Three trials randomized individual students to the intervention or control condition [30–32]. In the remaining trials randomization was completed at the level of the cluster; the unit of randomization was the school in 6 trials [28,33–37] and the classroom in 16 trials [20,38–51]. Three studies [42,48,52] evaluated outcomes after only one session of the intervention, while the remaining 22 implemented the intervention weekly with an intervention length ranging from 2 weeks to 3 years. Most intervention sessions lasted approximately10-30 minutes and were implemented 3 times per week.

### Risk of bias in included studies

The overall risk of bias in each included study are presented in Table 1. Only one study had low risk of bias and the remaining studies were judged as either having some concerns or high risk of bias. Risk of bias arising due to the randomization process was most problematic as it was rated as at least some concern for bias in 14 of the 25 studies; this was partly due to poor reporting of the randomization process leading to uncertainty regarding allocation concealment, as well as imbalanced baseline factors suggesting there may have been an issue with the randomization process.

**Table 1:** Risk of bias assessment.

Author	Year	Design type	Outcome	Randomization process	Bias arising from the timing of identification and recruitment	Bias due to deviations from intended interventions	Bias due to missing outcome data	Bias in measurement of the outcome	Bias in selection of the reported result	*Overall Risk of Bias*
Beck et al.	2016	RCT (Cluster)	Academic performance, EF, fluid intelligence,	low risk	low risk	some concerns	low risk	low risk	low risk	*Some concern*
de Greeff & Mullender-Wijnsma	2016	RCT (Cluster)	Academic performance, EF	low risk	low risk	some concerns	low risk	low risk	low risk	*Some concerns*
Donnelly & Szabo-Reed	2017	RCT (Cluster)	Academic performance,	some concerns	low risk	some concerns	low risk	low risk	low risk	*Some concerns*
			Time on task				some concerns	high risk		*High risk*
Fedewa et al.	2015	RCT (Cluster)	Academic performance, fluid intelligence	some concerns	low risk	some concerns	low risk	low risk	low risk	*Some concern*
Grieco et al.	2016	RCT (Cluster)	Time on task	low risk	low risk	low risk	low risk	low risk	low risk	*Low risk*
Kirk et al.	2014	RCT (Cluster)	Academic performance	some concerns	low risk	low risk	low risk	low risk	low risk	*Some concerns*
Mavilidi et al.	2016	RCT (cluster)	Academic performance	some concerns	low risk	some concern	low risk	low risk	low risk	*Some concerns*
			enjoyment				low risk	low risk		
Mavilidi et al.	2015	RCT (Cluster)	Academic performance	some concerns	low risk	some concern	some concerns	low risk	low risk	*Some concern*
Norris et al.	2015	RCT (Cluster)	Academic performance	low risk	low risk	low risk	low risk	low risk	low risk	*Low risk*
			enjoyment				low risk	high risk		*High risk*
Riley et al.	2016	RCT (Cluster)	Academic performance	low risk	low risk	some concern	low risk	low risk	low risk	*Some concern*
			Time on task				low risk	high risk		*High risk*
			enjoyment				low risk	high risk		*High risk*
Riley et al.	2015	RCT (Cluster)	enjoyment	low risk	low risk	some concern	low risk	high risk	low risk	*High risk*
			Time on task				low risk	high risk		*High risk*
Sun et al.	2016	RCT	Academic performance	some concerns	n/a	low risk	low risk	low risk	low risk	*Some concerns*
			enjoyment				low risk	high risk		*High risk*
Toumpaniari et al.	2015	RCT (Cluster)	Academic performance	some concerns	low risk	some concerns	low risk	low risk	low risk	*Some concerns*
			enjoyment				low risk	low risk		
Stewart et al.	2016	RCT (Cluster)	Academic performance	some concerns	low risk	some concerns	low risk	low risk	low risk	*Some concern*
Donnelly et al.	2009	RCT (Cluster)	Academic performance	some concerns	low risk	some concerns	some concerns	low risk	low risk	*Some concerns*
Reed et al.	2010	RCT (Cluster)	Academic performance, fluid intelligence	low risk	low risk	some concerns	low risk	low risk	high risk	*High risk*
									low risk	*Some concerns*
Bartholomew et al.	2018	RCT (Cluster)	Time on task	low risk	low risk	low risk	low risk	high risk	low risk	*High risk*
Mavilidi et al.	2018	RCT (Cluster)	Academic performance	some concerns	low risk	some concerns	some concerns	some concerns	low risk	*Some concerns*
			Enjoyment				some concerns	low risk		
Mavilidi et al.	2017	RCT (Cluster)	Academic performance	low risk	low risk	some concerns	some concerns	some concerns	low risk	*Some concerns*
			Enjoyment				some concerns	low risk		
Raney et al.	2017	RCT (Cluster)	Academic performance	low risk	low risk	high risk	low risk	high risk	low risk	*High risk*
			Time on task				low risk	high risk	high risk	
Eloffsson et al.	2018	RCT	Academic performance	High risk	n/a	Low risk	Low risk	Low risk	Low risk	High risk
Have et al.	2018	RCT (cluster)	Academic performance	Some concern	Low risk	Low risk	Some concern	Low risk	Low risk	Some concerns
Hraste et al.	2018	RCT	Academic performance	High risk	n/a	Some concerns	Low risk	Low risk	low risk	High risk
Mavilidi et al.	2018b	RCT (cluster)	Academic performance, EF	High risk	Low risk	Some concerns	Low risk	Low risk	Low risk	High risk
			Time on task					High risk		
Norris et al.	2018	RCT (cluster)	Time on task	Low risk	Low risk	Low risk	Some concern	High risk	Low risk	High risk

RCT: Randomized controlled trial

### Effects of intervention

The following results are robust to changes in imputed ICCs values (see Table A and B in S1 File).

#### Academic performance

**Fig 2:**
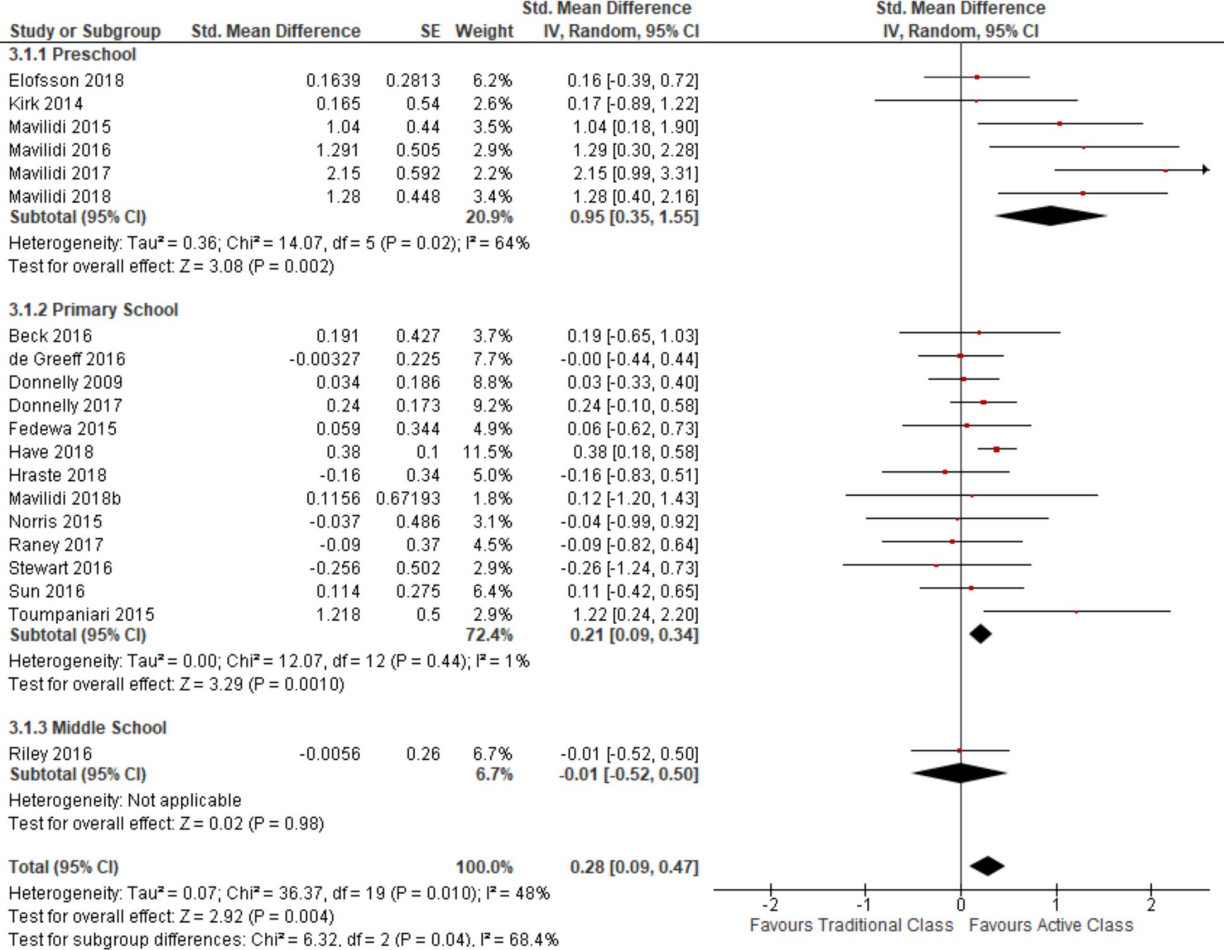
Overall academic performance by age subgroups.

Pooled estimates of all measures of academic performance from 20 studies of 842 (cluster adjusted) participants shows a small increase in performance on academic tests following an active classroom intervention compared to a traditional sedentary classroom (SMD = 0.28, 95% confidence interval (CI) range from 0.09 to 0.47 (See Fig 2). The overall I^2^ = 48%, indicating moderate to substantial heterogeneity. Sensitivity analyses excluding studies with imbalanced academic performance scores at baseline shows a slightly larger magnitude however lower precision of effect (See Figure G in S1 File). Sensitivity analyses examining only studies with a low risk of bias due to randomization show smaller effects and the CI incudes possible harm and no effect (See Figure H in S1 File).

Six studies were conducted in preschool or childcare centres with children (cluster adjusted n = 122) between the ages of 3 and 4 years; the pooled estimate shows an increase in academic performance (SMD = 1.05) with CI excluding no effect following active classroom lessons compared to traditional classrooms. The pooled estimate from ten studies looking at children in primary school (cluster adjusted n = 666) shows a smaller increase in academic performance (SMD = 0.21), however still excludes no effect of the intervention. Only one study examined children in middle school (cluster adjusted n = 63 and the estimate of effect showed a slight decrease in academic performance (SMD = -0.01), with a CI that includes no effect and possible benefits of active classrooms. Estimates of heterogeneity decreased in the preschool and primary school groups; the X^2^ test of subgroup differences was statistically significant indicating the effects differ across the population subgroups (See Fig 2).

Statistical tests of subgroup differences by academic subject could not be performed because many studies reported the effects of their intervention on multiple different subject types. Therefore, the effects of the intervention on each subject are presented separately (See Table 2 and Figures I-N in S1 File). Pooling nine studies shows a small increase in math scores of active classrooms (SMD = 0.08) however the 95% CI includes small possible harm and no effect. Pooled effects of six studies show similarly small and imprecise improvement in reading performance. Spelling performance was only evaluated in four studies and results are imprecise: the range of effect shows possible small harm, no effect, and benefits. Effects on language were only evaluated in two studies and results show increases in language performance with CI excluding no effect.

**Table 2:** Summary subgroup analyses.

Outcome or Subgroup Title	No. of studies	No. of participants	Statistical Method	Effect Size (95% CI)
Academic Performance Overall	20	842	Std. Mean Difference (IV, Random, 95% CI)	0.28 (0.09 to 0.47)
1.1.1Preschool	6	122	Std. Mean Difference (IV, Random, 95% CI)	0.95 (0.35 to 1.55)
1.1.2 Primary School	13	666	Std. Mean Difference (IV, Random, 95% CI)	0.21 (0.09 to 0.34)
1.1.3 Middle School	1	63	Std. Mean Difference (IV, Random, 95% CI)	-0.01 (-0.52 to 0.50)
1.2.1 Math	11	526	Std. Mean Difference (IV, Random, 95% CI)	0.08 (-0.09 to 0.26)
1.2.2 Reading	6	401	Std. Mean Difference (IV, Random, 95% CI)	0.04 (-0.16 to 0.24)
1.2.3 Spelling	4	341	Std. Mean Difference (IV, Random, 95% CI)	0.19 (-0.02 to 0.40)
1.2.4 Language	2	43	Std. Mean Difference (IV, Random, 95% CI)	1.07 (0.42 to 1.72)
1.2.5 Geography	1	19	Std. Mean Difference (IV, Random, 95% CI)	0.99 (0.02 to 1.96)
1.2.6 Science	3	94	Std. Mean Difference (IV, Random, 95% CI)	0.57 [-0.46, 1.61]
2. Executive Function	3	112	Std. Mean Difference (IV, Random, 95% CI)	-0.04 (-0.41 to 0.34)
3. Fluid Intelligence	2	61	Mean Difference (IV, Random, 95% CI)	0.38 (-3.21 to 3.96)
4. Time on Task	7	541	Std. Mean Difference (IV, Random, 95% CI)	0.37 (0.06 to 0.68)
4.1 Primary School	6	480	Std. Mean Difference (IV, Random, 95% CI)	0.41 (0.03 to 0.79)
4.2 Middle School	1	61	Std. Mean Difference (IV, Random, 95% CI)	0.32 (-0.19 to 0.83)
5. Enjoyment	6	148	Std. Mean Difference (IV, Random, 95% CI)	0.68 (0.35 to 1.02)
5.1 Preschool	3	61	Std. Mean Difference (IV, Random, 95% CI)	0.85 (0.32 to 1.38)
5.2 Primary School	3	87	Std. Mean Difference (IV, Random, 95% CI)	0.58 (0.14 to 1.01)

Std: Standardized; IV: Inverse Variance; CI: Confidence Interval

#### Cognitive abilities

**Fig 3:**
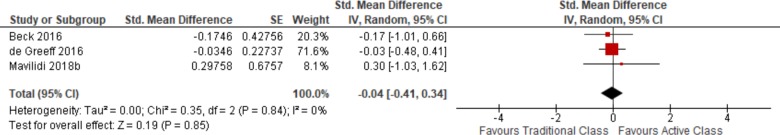
Executive function.

Three studies provided data on effects of active classrooms on EFs, including measures of inhibition, cognitive flexibility, and verbal and visual working memory (See Table 2 and Fig 3). Beck et al.[38] reported several indices resulting from a single test of EF; the interference accuracy score and interference response time were extracted and combined because this is widely accepted to be the most valid index of inhibition [53,54]. de Greeff et al.[39] applied four different tests of EF, an average SMD was computed as the estimate of effect for this study. Mavilidi [50] reported log transformed results from an inhibition task and response time and accuracy scores from a working memory task. The log-transformed scores were assumed to be skewed and therefore not appropriate to include in the meta-analysis and the working memory scores were combined. Pooled estimates show small negative effects of active classrooms compared to traditional classrooms on measures of EF (SMD = -0.04), however the CI include no effect and possible benefit. Two other studies used Raven’s Standard Progressive matrices to measure fluid intelligence and found close to no effect of active classrooms (See Table 2 and Figure O in S1 File). Subgroup and sensitivity analyses could not be computed for this outcome because there were too few studies.

#### Attention

**Fig 4:**
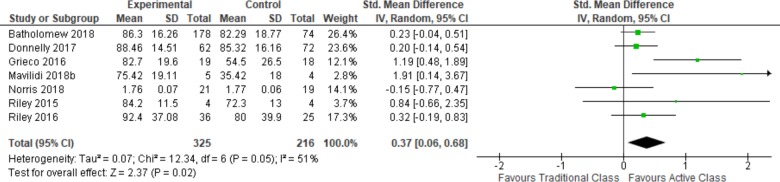
Attention measured as “Time on Task”.

Data from seven studies were available to estimate the effect of active classrooms on time on task compared to traditional classrooms (See Table 2 and Fig 4). Pooled estimates show increases in time spent on task following active classrooms with a confidence interval that excludes no effect.

#### Enjoyment

**Fig 5:**
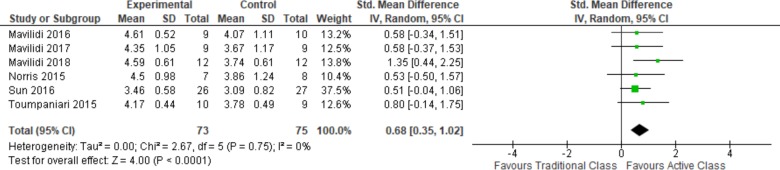
Enjoyment.

Six studies measured students’ enjoyment of active classrooms compared to traditional classrooms, primarily using single-item pictorial Likert scales. Pooled estimates consistently show higher levels of enjoyment of active classrooms and the 95% CI excludes no effect (See Table 2 and Fig 5).

Overall, the quality of evidence of the impact of active classrooms on measures of included educational outcomes is low given the risk of bias of each study and serious imprecision of the pooled estimates. Publication bias was not detected as demonstrated by the funnel plot (See Figure P in S1 File).

## Discussion

### Summary of main results and certainty of evidence

The emergence of this intervention has led to the fast rise in available evidence evaluating active classrooms and their impact on a variety of important educational outcomes. The inclusion of all available eligible trials seems to suggest that integrating PA into academic classrooms may slightly increase academic achievement, time on task, and enjoyment compared to the traditional sedentary lessons. This effect may be slightly larger in preschool aged children than in primary or middle school aged children. Our current best available evidence for the effects of active classrooms on cognitive abilities is uncertain and ranges from having a medium negative effect to a medium positive effect.

### Overall completeness and applicability of evidence

Our review suggests that while there has been a surge in the volume of randomised trials completed and published, there are few studies that are adequately powered, employ appropriate methods of randomization, measure a wide range of important student outcomes (including student fatigue and incidence of injury), and include students across the full spectrum of the school-age. The primary limiting factors of the quality of evidence are imprecision and risk of bias. Given the nature of the intervention, cluster RCTs are the ideal study design for evaluation as they avoid issues of contamination; however very few trials were able to appropriately power their samples to account for the within-cluster variance and as such generated wide intervals of effect. This issue was further emphasized in consideration of the lack of reporting of ICC values within individual studies. Adequate power is a particularly relevant consideration for this research question given that the comparator is an ‘active’ control (i.e., they are still engaged in an academic lesson designed to improve learning) and therefore we may expect small but important effects. There was generally at least some concern or higher in terms of risk of bias of each study, especially due to the randomization process. Seven studies [30,33,34,47] showed baseline imbalances in the primary outcome, however a sensitivity analyses showed that these studies did not substantially bias the overall effect of the intervention. Measurement of the outcomes and attrition, however, appeared to be well done in a majority of the included studies, as they were assessed as having low risk of bias. These strengths highlight the tremendous value of conducting school-based trials from a pragmatic perspective. Especially with regards to measuring academic outcomes, these are easily implemented within the school day as they are familiar to both students and teachers. Furthermore, given that the academic tests evaluate knowledge, it is unlikely that awareness of intervention status would affect children’s performance on tests, thus minimizing systematic error. Most included studies were able to measure academic performance with low risk of bias. Additionally, by completing the assessments in-school, attrition bias is limited because missing data is likely due to factors unrelated to the study (e.g. school absence due to illness). Therefore, only a few studies had some concerns for bias, with the remaining at low risk of attrition bias. Nonetheless, school-based RCTS are difficult to design and conduct given the range of involved stakeholders and practical concerns. A randomized controlled study necessitates substantial support and investment from both the schools and the larger community. Ensuring community engagement, providing sufficient support to school staff, limiting disruptions to class time, and ensuring open communication across all stakeholder groups, including parents, teachers, and children are examples of only some of the obstacles that need to be overcome in a single school-based trial [55,56]. Notwithstanding these practical barriers inherent in school-based studies, methodological and reporting standards could be improved substantially.

Furthermore, the current quality of evidence for secondary outcomes of cognitive abilities, attention, and enjoyment is very low; this is mostly due to the low number of studies including these measures. We could not perform meaningful subgroup analyses on these secondary outcomes; therefore, we are uncertain of whether these summary estimates of negligible effects including both possible harm and benefit may be different in defined subgroups.

Lastly, the evidence is largely limited in its ability to explore process outcomes, such as fidelity to and feasibility of the intervention, or mechanisms leading to improved educational outcomes. A major barrier to implementation is likely time to plan the lesson, given that the intervention is intentional integration of physical activity into a classroom lesson, which deviates from the typical sedentary classroom lesson. Naylor and colleagues found that time to prepare and deliver physical activity session was a key element the influenced teachers when implementing PA interventions [12]. Therefore, more translational research is needed to identify strategies to minimize time barriers to ensure feasibility and sustainability of physically active classrooms. Furthermore, the evidence is limited in its ability to provide meaningful understanding of potential mechanisms and recommendations on the optimal dose, intensity, and duration of physically active lessons. More research is required to provide mechanistic evidence and precise estimates of effects across varying durations and intensities.

### Potential biases in the review process

We restricted the eligibility of studies by language, however this led to the exclusion of only one trial and as such it is unlikely to substantially change the results of the analysis. This review required making assumptions about the correlation within clusters. Therefore, we used external sources to identify cluster-specific ICCs to calculate the effective sample sizes for each study and in many cases this substantially reduced the total sample size. Sensitivity analyses examining larger and smaller estimates of ICCs, however, showed similar magnitude and precision of effect suggesting our results are robust to various intra-cluster correlations. Lastly, we decided to restrict the comparison to outcomes measured immediately following the intervention completion to minimize multiple comparison issues; as such we are unable to comment on the long-term impacts of active classrooms.

### Agreements and disagreements with other studies or reviews

The results of the current review are in agreement with the conclusions drawn by Norris et al. in their 2015 review [18] that while the evidence base was small, there is a positive association between physically active lessons and educational outcomes. Our results also agree with reviews and meta-analyses of the effects of non-academic PA interventions on educational outcomes. Singh et al. in 2012 [57] reviewed both observational and intervention studies and found a positive effect of PA on measures of academic performance. Lees et al.[58] conducted a systematic review of randomized trials examining the cognitive effect of aerobic exercise and similar to our current review found either small benefits to cognition or no effects on measures of cognition and academic achievement. Finally, our results align with those reported in the review examining classroom-based PA interventions in general, showing positive results on academic-related outcomes (e.g., standardized test scores) [19].

### Conclusions

This review presents results on the impact of active classrooms on academic achievement, however the effect of this review will largely be to encourage the conduct of adequately powered, methodologically sound trials further evaluating the full effect of active classrooms on student-important outcomes.

## Supporting information

S1 File Search Strategy and Additional Analyses. Figure A. PyschINFO Search—March 3, 2017; Figure B. Updated PyschINFO Search–December 3, 2017; Figure C. Updated PyschINFO Search–February 5, 2019; Figure D. ERIC Search–March 3, 2017; Figure E. Updated ERIC Search–December 3, 2017; Figure F. Updated ERIC Search–February 5, 2019; Figure G. Overall Academic Performance; Balanced baseline performance scores sensitivity analysis; Figure H. Overall Academic Performance; Risk of Bias (Due to randomization) Sensitivity Analysis; Figure I. Academic Performance: Math; Figure J. Academic Performance: Reading; Figure K. Academic Performance: Spelling; Figure L. Academic Performance: Language; Figure M. Academic Performance: Geography; Figure N. Academic Performance: Science; Figure O. Fluid Intelligence; Figure P. Funnel Plot for Studies assessing the Effect of Overall Academic Performance; Table A. Summary of intervention effects with school-level ICC = 0.05 and class-level ICC = 0.17; Table B. Summary of intervention effects with school-level ICC = 0.15 and class-level ICC = 0.27.
(DOCX)

S2 File PRISMA checklist. (DOC)
