# Supplementary material for: A systematic review and meta-analysis on the effects of physically active classrooms on educational and enjoyment outcomes in school age children
Source: PLoS One. 2019 Jun 25;14(6):e0218633. doi: 10.1371/journal.pone.0218633 (PMC6592532; doi:10.1371/journal.pone.0218633)
Supplement: S1 File — Figure A. PyschINFO Search—March 3, 2017; Figure B. Updated PyschINFO Search–December 3, 2017; Figure C. Updated PyschINFO Search–February 5, 2019; Figure D. ERIC Search–March 3, 2017; Figure E. Updated ERIC Search–December 3, 2017; Figure F. Updated ERIC Search–February 5, 2019; Figure G. Overall Academic Performance; Balanced baseline performance scores sensitivity analysis; Figure H. Overall Academic Performance; Risk of Bias (Due to randomization) Sensitivity Analysis; Figure I. Academic Performance: Math; Figure J. Academic Performance: Reading; Figure K. Academic Performance: Spelling; Figure L. Academic Performance: Language; Figure M. Academic Performance: Geography; Figure N. Academic Performance: Science; Figure O. Fluid Intelligence; Figure P. Funnel Plot for Studies assessing the Effect of Overall Academic Performance; Table A. Summary of intervention effects with school-level ICC = 0.05 and class-level ICC = 0.17; Table B. Summary of intervention effects with school-level ICC = 0.15 and class-level ICC = 0.27. (DOCX) [file pone.0218633.s001.docx]

S1 File

**Figure A. PyschINFO Search - March 3, 2017**

| 1. physical activity.ti,ab. |
| --- |
| 2. activit*.ti,ab. |
| 3. exercise.ti,ab. |
| 4. class*.ti,ab. |
| 5. lesson*.ti,ab. |
| 6. learning*.ti,ab. |
| 7. child*.ti,ab. |
| 8. young*.ti,ab. |
| 9. 1 or 2 or 3 |
| 10. 4 or 5 or 6 |
| 11. 7 or 8 |
| 12. 9 and 10 and 11 |
| 13. limit 12 to yr="2014 -Current" |

**Figure B. Updated PyschINFO Search – December 3, 2017**

| 1. physical activity.ti,ab. |
| --- |
| 2. activit*.ti,ab. |
| 3. exercise.ti,ab. |
| 4. class*.ti,ab. |
| 5. lesson*.ti,ab. |
| 6. learning*.ti,ab. |
| 7. child*.ti,ab. |
| 8. young*.ti,ab. |
| 9. 1 or 2 or 3 |
| 10. 4 or 5 or 6 |
| 11. 7 or 8 |
| 12. 9 and 10 and 11 |
| 13. limit 12 to yr="2014 -Current" |
| 14. limit 13 to yr="2017 -Current" |

**Figure C. Updated PyschINFO Search – February 5, 2019**


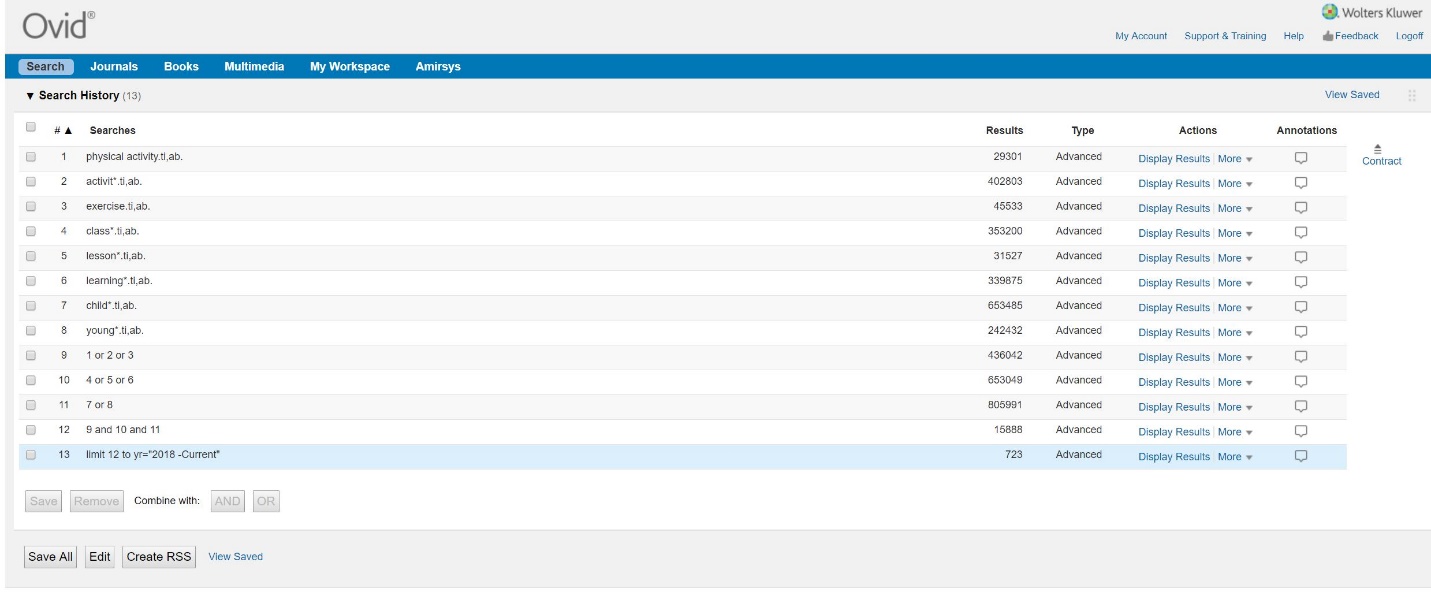


**Figure D. ERIC Search – March 3, 2017**


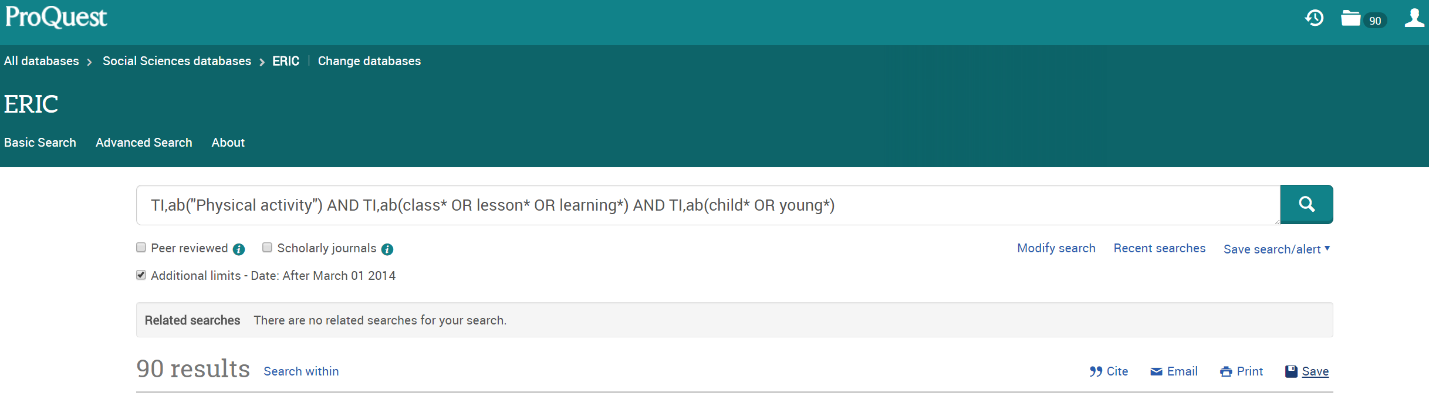


**Figure E. Updated ERIC Search – December 3, 2017**


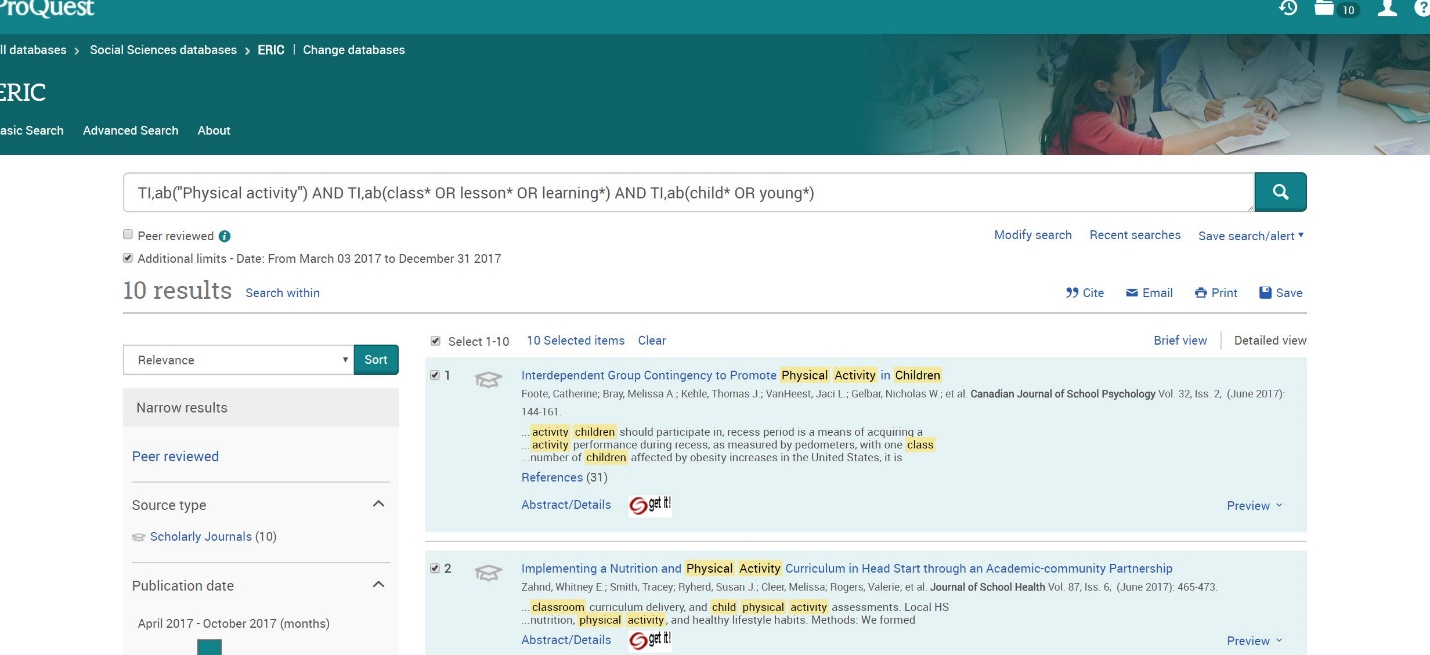


**Figure F. Updated ERIC Search – February 5, 2019**


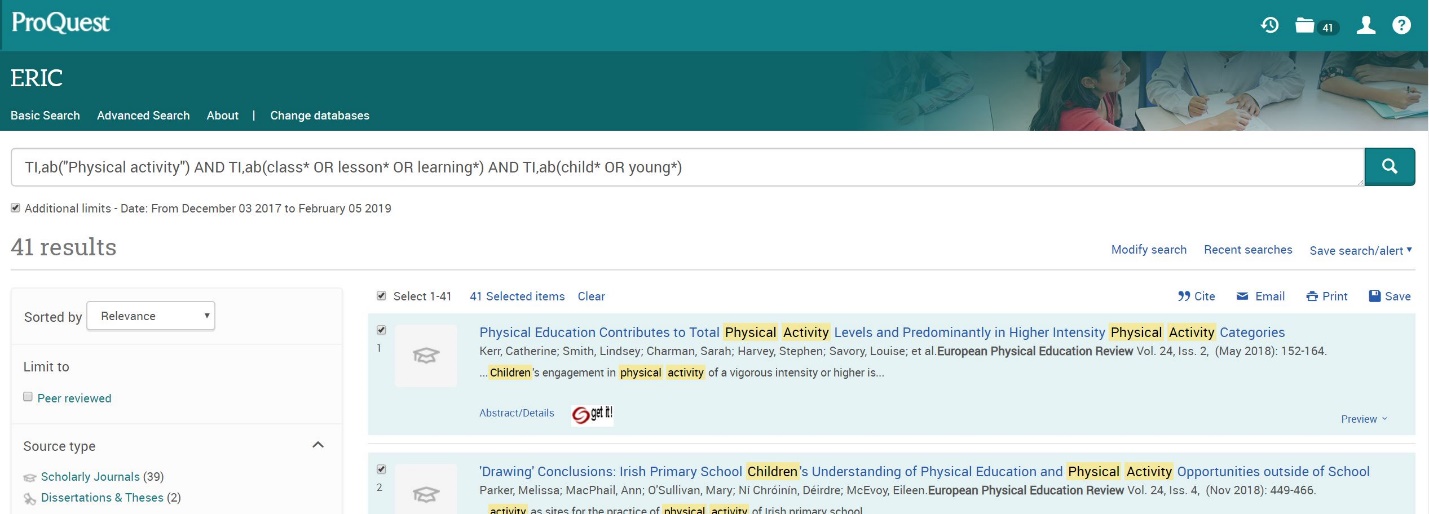


Additional Analyses

**Figure G. Overall Academic Performance; Balanced baseline performance scores sensitivity analysis**

**
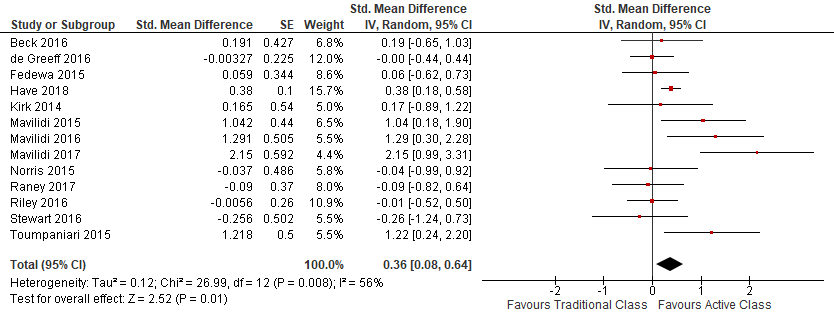
**

**Figure H. Overall Academic Performance; Risk of Bias (Due to randomization) Sensitivity Analysis**

**
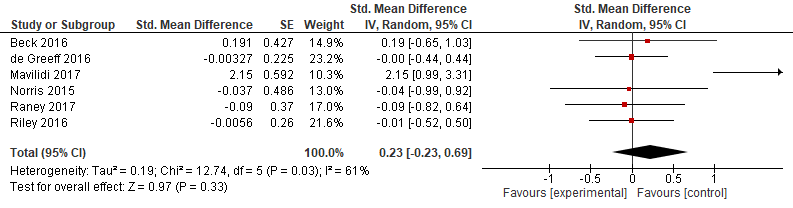
**

**Figure I. Academic Performance; Subject Subgroups*; Math**


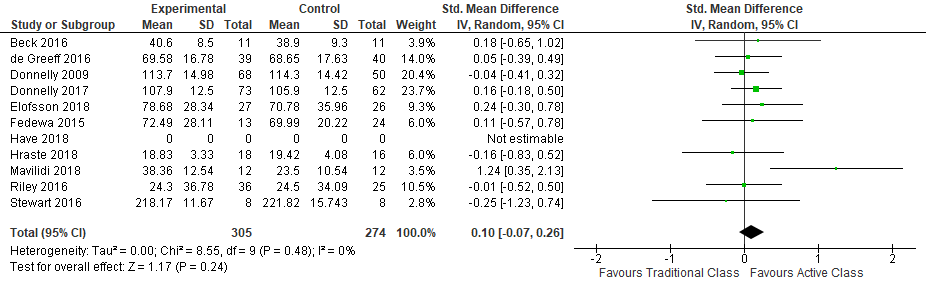


Notes: “Rote Counting” outcome data was entered for Elofsson 2018; sensitivity analyses using data from each of the 8 math outcomes reported in Elofsson 2018 shows a range of point estimates from -0.01 to 0.19. Individual group means were not reported in Have 2018, therefore this data is not included in this analysis.

**Figure J. Academic Performance; Subject Subgroups*; Reading**


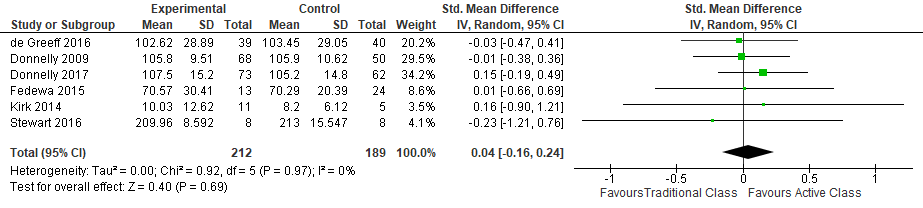


**Figure K. Academic Performance; Subject Subgroups*; Spelling**

**
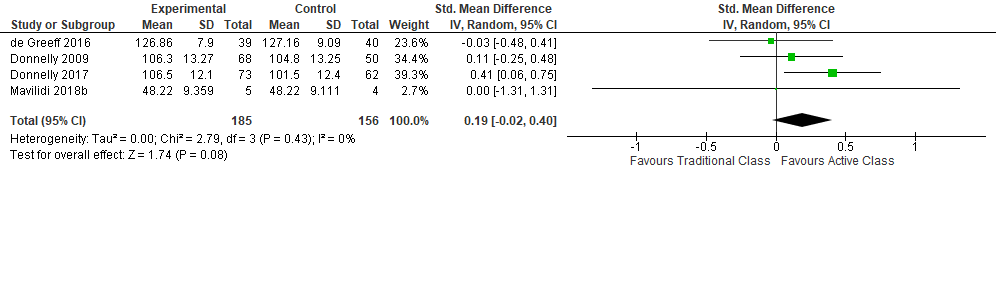
**

**Figure L. Academic Performance; Subject Subgroups*; Language**

**
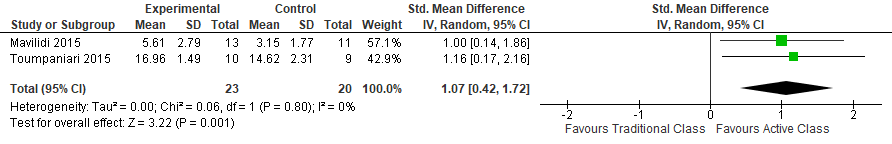
**

**Figure M. Academic Performance; Subject Subgroups*; Geography**


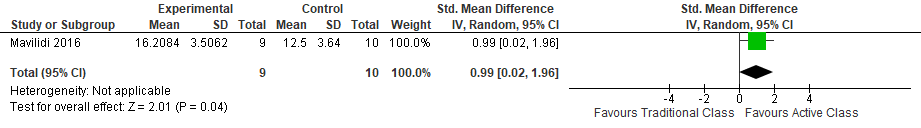


**Figure N. Academic Performance; Subject Subgroups*; Science**


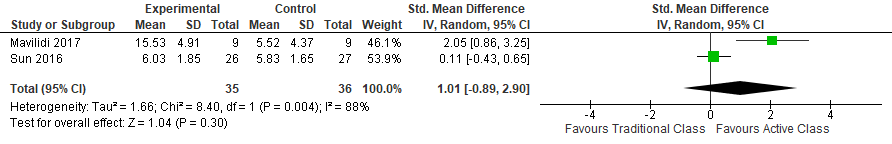


***** Statistical tests of subgroups differences by subject could not be performed because many studies reported the effects of their intervention on multiple different subject type. Therefore, to avoid issues of multiplicity by running them as subgroups in RevMan, the effects of the intervention on each subject are presented separately.

**Figure O. Fluid Intelligence**

**
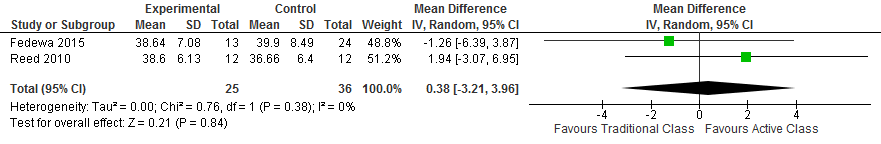
**

**Figure P. Funnel Plot for Studies assessing the Effect of Overall Academic Performance**


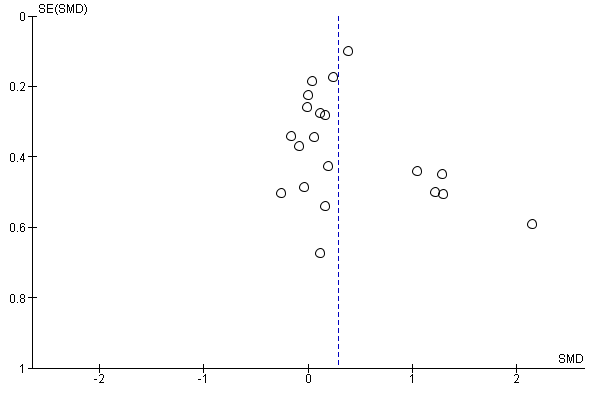


Table A. Summary of intervention effects with school-level ICC=0.05 and class-level ICC=0.17

| Outcome or Subgroup Title | No. of studies | No. of participants | Statistical Method | Effect Size |
| --- | --- | --- | --- | --- |
|  |  |  |  |  |
| 1. Academic Performance Overall | 20 | 1156 | Standardized Mean Difference (IV, Random, 95% CI, School ICC=0.05, Class ICC=0.17) | 0.32 [0.12, 0.52] |
| 1.1.1 Preschool | 6 | 195 | Standardized Mean Difference (IV, Random, 95% CI, School ICC=0.05, Class ICC=0.17) | 1.08 [0.39, 1.78] |
| 1.1.2 Primary School | 13 | 916 | Standardized Mean Difference (IV, Random, 95% CI, School ICC=0.05, Class ICC=0.17) | 0.18 [0.05, 0.32] |
| 1.1.3 Middle School | 1 | 98 | Standardized Mean Difference (IV, Random, 95% CI, School ICC=0.05, Class ICC=0.17) | -0.01 [-0.41, 0.40] |
| 1.5.1 Math | 11 | 835 | Standardized Mean Difference (IV, Random, 95% CI, School ICC=0.05, Class ICC=0.17) | 0.08 [-0.07, 0.22] |
| 1.5.2 Reading | 6 | 578 | Standardized Mean Difference (IV, Random, 95% CI, School ICC=0.05, Class ICC=0.17) | 0.05 [-0.11, 0.22] |
| 1.5.3 Spelling | 4 | 474 | Standardized Mean Difference (IV, Random, 95% CI, School ICC=0.05, Class ICC=0.17) | 0.19 [-0.03, 0.42] |
| 1.5.4 Language | 2 | 49 | Standardized Mean Difference (IV, Random, 95% CI, School ICC=0.05, Class ICC=0.17) | 1.07 [0.47, 1.68] |
| 1.5.5 Geography | 1 | 23 | Standardized Mean Difference (IV, Random, 95% CI, School ICC=0.05, Class ICC=0.17) | 1.08 [0.19, 1.96] |
| 1.5.6 Science | 3 | 104 | Standardized Mean Difference (IV, Random, 95% CI, School ICC=0.05, Class ICC=0.17) | 0.61 [-0.46, 1.67] |
| 2. Executive Function | 3 | 136 | Standardized Mean Difference (IV, Random, 95% CI, School ICC=0.05, Class ICC=0.17) | -0.04 [-0.38, 0.31] |
| 3. Fluid Intelligence | 2 | 98 | Mean Difference (IV, Random, 95% CI, School ICC=0.05, Class ICC=0.17) | 0.07 [-3.02, 3.16] |
| 4. Time on Task | 7 | 911 | Standardized Mean Difference (IV, Random, 95% CI, School ICC=0.05, Class ICC=0.17) | 0.44 [0.14, 0.74] |
| 3.1.1 Primary School | 6 | 813 | Standardized Mean Difference (IV, Random, 95% CI, School ICC=0.05, Class ICC=0.17) | 0.49 [0.13, 0.86] |
| 3.1.2 Middle School | 1 | 98 | Standardized Mean Difference (IV, Random, 95% CI, School ICC=0.05, Class ICC=0.17) | 0.32 [-0.08, 0.73] |
| 5. Enjoyment | 6 | 163 | Standardized Mean Difference (IV, Random, 95% CI, School ICC=0.05, Class ICC=0.17) | 0.69 [0.37, 1.01] |
| 2.2.1 Preschool | 3 | 72 | Standardized Mean Difference (IV, Random, 95% CI, School ICC=0.05, Class ICC=0.17) | 0.84 [0.35, 1.34] |
| 2.2.2 Primary School | 3 | 91 | Standardized Mean Difference (IV, Random, 95% CI, School ICC=0.05, Class ICC=0.17) | 0.58 [0.15, 1.00] |

Table B. Summary of intervention effects with school-level ICC=0.15 and class-level ICC=0.27

| Outcome or Subgroup Title | No. of studies | No. of participants | Statistical Method | Effect Size |
| --- | --- | --- | --- | --- |
|  |  |  |  |  |
| 1. Academic Performance Overall | 20 | 682 | Standardized Mean Difference (IV, Random, 95% CI, School ICC=0.15, Class ICC=0.27) | 0.30 [0.10, 0.50] |
| 1.1.1 Preschool | 6 | 147 | Standardized Mean Difference (IV, Random, 95% CI, School ICC=0.15, Class ICC=0.27) | 0.97 [0.36, 1.58] |
| 1.1.2 Primary School | 13 | 542 | Standardized Mean Difference (IV, Random, 95% CI, School ICC=0.15, Class ICC=0.27) | 0.23 [0.09, 0.36] |
| 1.1.3 Middle School | 1 | 46 | Standardized Mean Difference (IV, Random, 95% CI, School ICC=0.15, Class ICC=0.27) | -0.01 [-0.60, 0.59] |
| 1.5.1 Math | 11 | 423 | Standardized Mean Difference (IV, Random, 95% CI, School ICC=0.15, Class ICC=0.27) | 0.09 [-0.11, 0.28] |
| 1.5.2 Reading | 6 | 312 | Standardized Mean Difference (IV, Random, 95% CI, School ICC=0.15, Class ICC=0.27) | 0.03 [-0.19, 0.26] |
| 1.5.3 Spelling | 4 | 269 | Standardized Mean Difference (IV, Random, 95% CI, School ICC=0.15, Class ICC=0.27) | 0.18 [-0.06, 0.42] |
| 1.5.4 Language | 2 | 40 | Standardized Mean Difference (IV, Random, 95% CI, School ICC=0.15, Class ICC=0.27) | 1.05 [0.38, 1.73] |
| 1.5.5 Geography | 1 | 19 | Standardized Mean Difference (IV, Random, 95% CI, School ICC=0.15, Class ICC=0.27) | 0.99 [0.02, 1.96] |
| 1.5.6 Science | 3 | 91 | Standardized Mean Difference (IV, Random, 95% CI, School ICC=0.15, Class ICC=0.27) | 0.58 [-0.48, 1.65] |
| 2. Executive Function | 3 | 95 | Standardized Mean Difference (IV, Random, 95% CI, School ICC=0.15, Class ICC=0.27) | -0.03 [-0.44, 0.37] |
| 3. Fluid Intelligence | 2 | 46 | Mean Difference (IV, Random, 95% CI, School ICC=0.15, Class ICC=0.27) | 0.52 [-3.58, 4.61] |
| 4. Time on Task - POP | 7 | 399 | Standardized Mean Difference (IV, Random, 95% CI, School ICC=0.15, Class ICC=0.27) | 0.36 [0.04, 0.68] |
| 4.1.1 Primary School | 6 | 354 | Standardized Mean Difference (IV, Random, 95% CI, School ICC=0.15, Class ICC=0.27) | 0.40 [0.00, 0.79] |
| 4.1.2 Middle School | 1 | 45 | Standardized Mean Difference (IV, Random, 95% CI, School ICC=0.15, Class ICC=0.27) | 0.32 [-0.28, 0.92] |
| 5. Enjoyment | 6 | 143 | Standardized Mean Difference (IV, Random, 95% CI, School ICC=0.15, Class ICC=0.27) | 0.68 [0.34, 1.02] |
| 5.1.1 Preschool | 3 | 61 | Standardized Mean Difference (IV, Random, 95% CI, School ICC=0.15, Class ICC=0.27) | 0.85 [0.32, 1.38] |
| 5.1.2 Primary | 3 | 82 | Standardized Mean Difference (IV, Random, 95% CI, School ICC=0.15, Class ICC=0.27) | 0.57 [0.12, 1.01] |
